# Fibroblast growth factor signaling in axons: from development to disease

**DOI:** 10.1186/s12964-023-01284-0

**Published:** 2023-10-16

**Authors:** Diogo Tomé, Marta S. Dias, Joana Correia, Ramiro D. Almeida

**Affiliations:** 1 Institute of Biomedicine, Department of Medical Sciences - iBiMED, University of Aveiro, Aveiro, Portugal; 2 CNC - Center for Neuroscience and Cell Biology, University of Coimbra, Coimbra, Portugal

**Keywords:** FGF, Signaling pathways, Axons, Presynaptic terminal, Axonal injury, Neuronal disorders

## Abstract

The fibroblast growth factor (FGF) family regulates various and important aspects of nervous system development, ranging from the well-established roles in neuronal patterning to more recent and exciting functions in axonal growth and synaptogenesis. In addition, FGFs play a critical role in axonal regeneration, particularly after spinal cord injury, confirming their versatile nature in the nervous system. Due to their widespread involvement in neural development, the FGF system also underlies several human neurological disorders. While particular attention has been given to FGFs in a whole-cell context, their effects at the axonal level are in most cases undervalued. Here we discuss the endeavor of the FGF system in axons, we delve into this neuronal subcompartment to provide an original view of this multipurpose family of growth factors in nervous system (dys)function.

Video Abstract

**Supplementary Information:**

The online version contains supplementary material available at 10.1186/s12964-023-01284-0.

## Introduction

The fibroblast growth factor (FGF) family are polypeptides that act through four highly conserved transmembrane tyrosine kinase receptors to elicit a range of context-dependent tissue and cellular outcomes, including patterning, morphogenesis, migration, survival and differentiation [1]. The expression of FGFs and their receptors is temporally and spatially regulated during neurodevelopment contributing to a plethora of effects in the nerve cell. FGF receptors (FGFRs) are generated by tissue-specific alternative splicing, leading to different ligand binding specificities [2]. FGFs engagement of the respective FGFRs triggers receptor dimerization and tyrosine kinase activation, resulting in autophosphorylation of the intracellular domain, recruitment and assembly of signaling complexes [3]. Although knowledge about FGF signaling in the nervous system has accumulated in recent years, we are lacking a focused review on the effects of the FGF family in axons. Here we propose to unravel how FGFs impact axonal biology. Firstly, we will describe the FGF ligands and their receptors, the signaling pathways and their modulators. Secondly, we will focus on the effects of this family of growth factors on axonal specification and growth, axonal guidance, presynaptic differentiation and synaptic function. Lastly, we will discuss how FGFs modulate axonal regeneration and how abnormal function underlies nervous system dysfunction.

### Fibroblast growth factor system

#### FGF ligands and their receptors

**Fig. 1:**
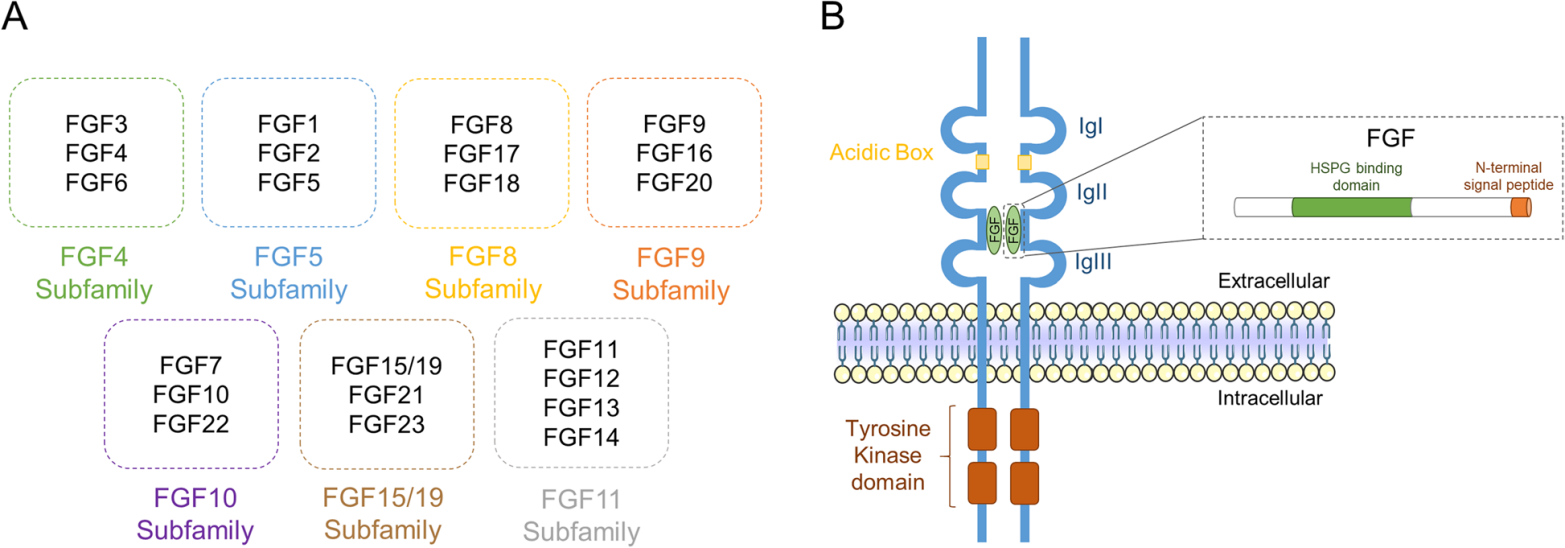
Fibroblast growth factor (FGF) phylogeny and receptor structure. **A** The FGFs subfamilies. The 22 FGF ligands are divided into 7 subfamilies according to their cofactors, binding specificity, sequence similarities and evolutionary relationships. **B** Schematic representation of the FGF-FGFR complex. This complex is composed of two receptor dimers and two FGFs. FGFRs (blue) are transmembrane proteins whose extracellular domain contains three immunoglobulin-like domains (IgI-IgIII) and an acidic box domain (yellow). Following a transmembrane α-helix, the intracellular domain is composed by a split tyrosine kinase domain (orange). The binding site for FGFs (green) comprises the region between the C-terminal portion of IgII and the N-terminal portion of IgIII. All FGFs include a heparan sulfate proteoglycan (HSPG) binding domain and the majority present a N-terminal signal peptide

The mammalian FGF family comprises 22 members, further divided into seven subfamilies based on interacting cofactors, binding and activation of FGFRs, sequence similarities and evolutionary relationships [3] (Fig. 1A). All FGFs present a heparan sulfate proteoglycan (HSPG) binding domain and most have cleavable N-terminal signal peptides and are secreted through the classical endoplasmic reticulum (ER)-Golgi secretory pathway [4]. Interestingly, the three members of the FGF9 subfamily (FGF9, FGF16 and FGF20) are efficiently secreted via the ER-Golgi pathway without an obvious signal peptide. Instead, this subfamily owns an atypical hydrophobic sequence that functions as a non-cleaved signal for transport into the ER [5]. On the other hand, the two FGF1 subfamily members (FGF1 and FGF2) lack a signal peptide and do not follow the conventional ER-Golgi secretory route but are readily exported from cells by direct translocation across the cell membrane. FGF1 secretion involves the formation of a specific multiprotein complex composed of synaptotagmin 1 and S100A13 [6], while FGF2 release involves the formation of pores across the cell membrane in a process dependent on the interaction with phosphatidylinositol-4,5-biphosphate (PIP_2_) [7]. Alternatively, FGF2 can also be secreted in exosomes that are then engulfed and internalized by the target cells [8]. Another subset of FGFs (FGF11 to FGF14) lack the ability to activate FGFRs and are not secreted from cells. Instead, they localize to the nucleus or interact with sodium [9] and calcium channels [10, 11] to modulate synaptic transmission and cardiac rhythm.

Most secreted FGFs function in a classic autocrine or paracrine fashion. They use HSPGs as binding partners, which stabilize and confer specificity to FGF ligand-receptor interaction, forming a ternary complex with FGFR. Additionally, HSPGs enhance FGFs resistance to proteolysis and control ligand diffusion, limiting the action of FGFs to their release site [12]. Conversely, the members of FGF15/19 subfamily (including FGF15/19, FGF21 and FGF23) act as endocrine factors and exhibit reduced heparan-binding affinity. This low affinity to bind HSPGs allows endocrine FGFs to diffuse from the release site into the circulation where they can act hormonally. Instead of using HSPGs as cofactors for receptor binding and activation, endocrine FGFs utilize members of the Klotho family [13]. These coreceptors not only enhance binding of endocrine FGFs to FGFR but also seem to inhibit the action of paracrine FGFs. The binding site for klotho coreceptors on FGFR partially overlaps with the binding site for ligands of the FGF8 subfamily, suggesting a reduced sensitivity of Klotho-expressing cells to these FGFs and possibly to other paracrine FGFs [14].

**Fig. 2:**
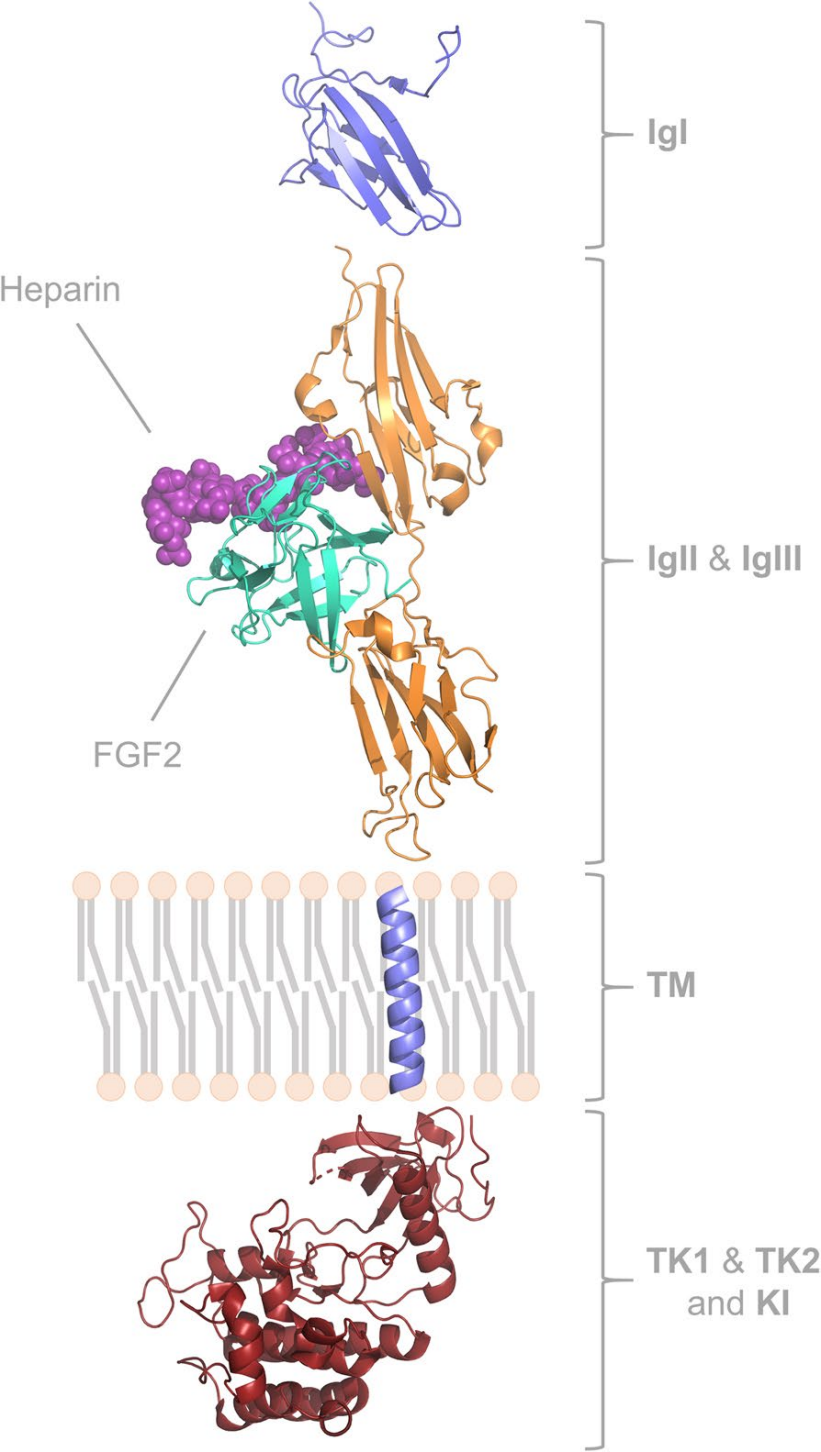
FGFR1-FGF2 complex protein structure. IgI, Immunoglobulin-like domain I (blue, PDB 2CR3); IgII & IgIII, Immunoglobulin-like domains II and III (orange, PDB 1FQ9); TM, Transmembrane domain (purple, AF-P11362-F1 *predicted structure)*; TK1 & TK2, Tyrosine kinase subdomains and KI, kinase insert (red, PDB 4UWY)

FGF signaling is transduced through a family of four FGFRs (FGFR1 to 4) in all vertebrates. They are single spanning transmembrane proteins whose extracellular domain is composed of three immunoglobulin-like domains (IgI-IgIII) and an unusual stretch of glutamate-, aspartate- and serine-rich sequence, termed the acid box domain. Following a transmembrane α-helix, the intracellular domain harbors a split tyrosine kinase domain. The region between the C terminal portion of IgII and the N-terminal portion of IgIII constitutes the FGF binding site, whereas the acid box domain is located between IgI and IgII (Fig. 1B). They interact with HSPGs via their IgII domain [15] (Fig. 2). The acid box domain is essential for the interaction with N-cadherin and neural cell adhesion molecule (NCAM) that require the tyrosine kinase activity of the FGFR to induce neuritogenesis [16, 17]. It also plays a key role in FGFR autoinhibition by electrostatically engaging the HSPGs binding site on the IgII domain. This interaction blocks HSPG and FGF binding to the receptor, serving as the first line of defense against excessive FGF signaling [18].

FGFRs are subjected to alternative splicing events that affect their biological function and are generally tissue specific. These events encompass the second half of the IgIII domain of FGFR1-3, creating IIIb and IIIc isoforms, which have different ligand binding specificities [19]. Only FGF1 can efficiently activate all receptor splice variants. However, it should be noted that the ligand binding specificity of the four FGFRs was established in cell culture assays [2, 20] (Table 1). Since HSPGs and other cofactors can modulate ligand-receptor interaction, the in vivo specificity of FGFRs can substantially diverge from in vitro observations. Additionally, a fifth related receptor, known as FGFRL1, can also bind FGFs, but lacks an intracellular kinase domain and might function as a negative regulator of FGF signaling [21].

**Table 1:** Ligand specificity of FGFRs

	***FGF4 Subfamily***			***FGF5 Subfamily***			***FGF8 Subfamily***			***FGF9 Subfamily***			***FGF10 Subfamily***			***FGF15/19 Subfamily***			***FGF11 Subfamily***			
	**3**	**4**	**6**	**1**	**2**	**5**	**8**	**17**	**18**	**9**	**16**	**20**	**7**	**10**	**22**	**15 / 19**	**21**	**23**	**11**	**12**	**13**	**14**
**FGFR1b**	**✓**			**✓**	**✓**							**✓**		**✓**	**✓**	**✓**	**✓**	**✓**				
**FGFR1c**		**✓**	**✓**	**✓**	**✓**	**✓**	**✓**	**✓**		**✓**						**✓**	**✓**	**✓**				
**FGFR2b**	**✓**			**✓**	**✓**					**✓**		**✓**	**✓**	**✓**	**✓**	**✓**	**✓**	**✓**				
**FGFR2c**		**✓**	**✓**	**✓**	**✓**	**✓**	**✓**	**✓**		**✓**	**✓**					**✓**	**✓**	**✓**				
**FGFR3b**				**✓**	**✓**					**✓**		**✓**				**✓**	**✓**	**✓**				
**FGFR3c**		**✓**		**✓**	**✓**		**✓**	**✓**	**✓**	**✓**	**✓**	**✓**				**✓**	**✓**	**✓**				
**FGFR4**		**✓**	**✓**	**✓**	**✓**		**✓**	**✓**	**✓**			**✓**	**✓**			**✓**	**✓**	**✓**				

#### Intracellular signaling pathways

**Fig. 3:**
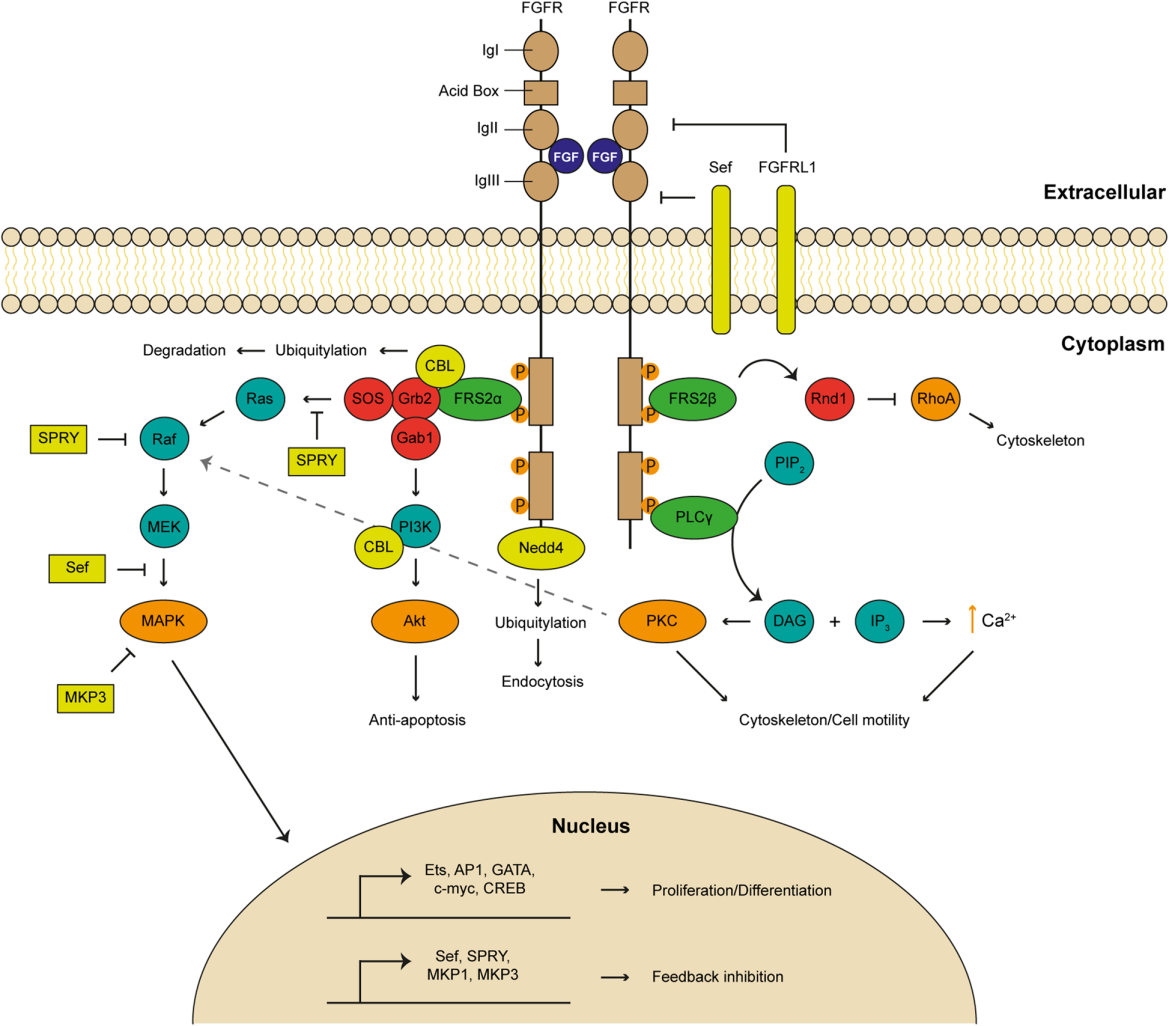
Intracellular signaling pathways activated downstream of fibroblast growth factor receptors (FGFRs). Binding of FGFs to FGFRs triggers receptor dimerization and tyrosine kinase activation, resulting in autophosphorylation of the intracellular domains of the receptor and recruitment of several adaptor proteins, which activate four key downstream pathways. The MAPK pathway involves the adaptor protein FRS2α that recruits Grb2 and SOS, resulting in the formation of a multiprotein complex that activates the Ras GTPase and the downstream targets Raf and MEK. This signaling cascade culminates in transcriptional activation of effectors and feedback inhibitors that mediate most of the developmental functions of FGFs. Grb2 can also recruit the adaptor protein Gab1, which activates the anti-apoptotic PI3K/Akt pathway. The PKC/calcium pathway is initiated by recruitment of PLCγ to phosphorylated tyrosines present in FGFR, leading to its activation and consequent formation of IP_3_ and DAG by hydrolysis of PIP_2_. While IP_3_ stimulates calcium release from intracellular stores, DAG activates PKC, events that promote the remodeling of cytoskeleton and cell membranes. PKC also reinforces the activation of the MAPK pathway by inducing RAF phosphorylation (dashed arrow). Activated FGFR also promotes the dissociation of Rnd1 from FRS2β, which in turn inhibits RhoA activity, leading to cytoskeletal rearrangements. Signaling can be negatively regulated at multiple levels by receptor internalization or the induction of negative regulators. The E3 ubiquitin ligases CBL and Nedd4 promote receptor degradation/turnover through ubiquitin-mediated mechanisms. CBL also mediates the degradation of PI3K attenuating the PI3K/Akt pathway. Proteins that antagonize FGFR signals can either interfere with ligand binding (FGFRL1 and Sef) or with intracellular signaling cascades, mainly the MAPK pathway (Sef, SPRY, MKP3). DAG, Diacylglycerol; FRS2, FGFR substrate; Grb2, Growth factor receptor-bound 2; Gab1, Grb2-associated binding protein 1; IP_3_, Phosphatidylinositol-3,4,5-triphosphate; MAPK, Mitogen-activated protein kinase; MKP3, MAPK phosphatase 3; PI3K, Phosphatidylinositol-3-kinase; PIP_2_, phosphatidylinositol-4,5-biphosphate; PKC, Protein kinase C; PLCγ, Phospholipase Cγ; SOS, Son of sevenless

Binding of FGFs to FGFRs induces receptor dimerization and increases kinase activity, leading to autophosphorylation of tyrosine residues in the intracellular domain of the receptor. Phosphorylated residues function as docking sites for adaptor proteins, which themselves may also be targeted for phosphorylation by activated FGFRs, resulting in the activation of multiple signaling pathways [22] (Fig. 3). The major FGFR kinase substrate is the adaptor protein FGFR substrate 2α (FRS2α) that upon phosphorylation promotes the recruitment of the adaptor proteins growth factor receptor-bound 2 (Grb2) and son of sevenless (SOS). The newly formed FRS2α-Grb2-SOS complex activates Ras GTPase and the downstream RAF, which in turn activates the mitogen-activated protein kinase (MAPK)/Erk pathway [23]. The MAPK/Erk signaling cascade is the pathway most commonly employed by FGFRs and it is important in mediating the proliferative effects of FGFs through the activation of several transcription factors such as Ets proteins, AP1, GATA proteins, c-myc and CREB [24]. Moreover, Grb2 can also recruit the adaptor protein Grb2-associated binding protein 1 (Gab1), which activates phosphatidylinositol-3-kinase (PI3K) resulting in activation of the anti-apoptotic Akt/protein kinase B (PKB) pathway [25].

Phospholipase Cγ (PLCγ) is another player involved in FGF signaling. Through its Src homology 2 (SH2) domain, PLCγ can also interact with phosphorylated tyrosines present in FGFR, independently of FRS2 presence [26]. This interaction leads to PLCγ activation and consequent formation of phosphatidylinositol-3,4,5-triphosphate (IP_3_) and diacylglycerol (DAG) by hydrolysis of PIP_2_. While IP_3_ stimulates calcium release from intracellular stores, DAG activates protein kinase C (PKC), events that mainly mediate cell motility through remodeling of cytoskeleton and cell membranes. This pathway has been implicated in the stimulation of neurite outgrowth in retinal ganglion cells (RGCs) by FGF2 [27]. Additionally, PKC activation partly reinforces the activation of the MAPK/Erk pathway due to RAF phosphorylation [24]. An additional pathway, involving the adaptor protein FRS2β and the small GTPases Rnd1 and RhoA, is also involved in cytoskeletal rearrangements. FGFR induces FRS2β phosphorylation causing the dissociation of Rnd1 from FRS2β, which in turn inhibits RhoA activity, mediating the effect of FGF on neurite outgrowth in PC12 cells [28]. Depending on the cellular context, other pathways such as the signal transducer and activator of transcription (STAT) pathway, are also activated in response to FGF-FGFR interaction [29].

FGFs and FGFRs can also regulate cellular events through their translocation to the nucleus. Alongside with FGF11 subfamily, FGF1, FGF2 and FGF3 bear a nuclear localization signal (NLS) that may guide these molecules to the cell nucleus [30]. Nuclear import of exogenous FGF1 involves the ER-protein LRRC59, a Ran GTPase and the ɑ- and β-importins, karyopherin-ɑ1 and karyopherin-β1 that recognize the NLS [31]. Transportation of FGF2 to the nucleus also requires karyopherin-ɑ1 and karyopherin-β1 but it is dependent on Translokin protein rather than LRRC59 [32]. Regarding FGFRs, several different mechanisms have been described to explain their nuclear translocation. Instead of being transported to the cell membrane, newly synthesized FGFRs remain in the cytosol and interact with FGF ligands that contain NLS, allowing their translocation to the nucleus by a β-importin-dependent mechanism [33]. Alternatively, membrane activated FGFRs may be internalized in vesicles and, instead of being recycled back to the cell membrane or degraded within lysosomes, are taken to the ER. Once in the ER, FGFRs may be released into the cytosol through Sec61 translocon, where they can interact with β-importin and be transported into the nucleus [30]. Another possible route for membrane-bound FGFRs to reach the nucleus upon activation is through cleavage of their intracellular domains by proteases such as Granzyme B or γ-secretase. These truncated functional variants of the FGFRs are then transported into the nucleus by a yet unknown mechanism [34, 35]. After reaching the nucleus, FGFs and FGFRs may regulate the expression of several genes, including genes involved in neuronal cell development and neurite outgrowth [36].

#### Modulators of FGF signaling

FGF signaling plays a vital role in a number of developmental and homeostatic processes. Dysfunction in its many players can lead to various human diseases, ranging from cancer to neurological conditions. Not surprisingly, FGF signaling is regulated at multiple levels to ensure a tight control of its level, spread and timing. As mentioned earlier, HSPGs are essential to modulate FGF-FGFR interaction and to regulate FGFs availability, but other factors present in the extracellular space might modulate FGF signaling as well. This is the case of FGF binding proteins (FGFBPs) that augment FGF signaling presumably by chaperoning FGFs through the extracellular matrix until they reach FGFRs. To date, FGFBPs have been found to bind and enhance the activity of FGF1 and FGF7 subfamilies [37, 38]. Besides their role in tumor growth, angiogenesis and wound healing [39], FGFBPs have also been implicated in nervous system development, maintenance and repair [40]. Additional extracellular modulators of FGF signaling have also been identified. These include the secreted glycoprotein anosmin-1 and the transmembrane proteins fibronectin-leucine-rich transmembrane protein 3 (FLRT3) and L1 cell adhesion molecule (L1CAM) that potentiate FGF signaling [41].

To prevent excessive downstream signaling from FGFRs, a number of mechanisms are put into action. Following activation, the FGF-FGFR complex is internalized, resulting in receptor degradation or recycling, through a mechanism that involves receptor ubiquitylation by the E3 ubiquitin ligases Nedd4 and CBL. While Nedd4 mediated-ubiquitylation appears to be required for receptor endocytosis [42], CBL is involved in receptor degradation. The latter forms a ternary complex with phosphorylated FRS2α and Grb2, promoting ubiquitination and degradation of both FGFR and FRS2α in response to FGF stimulation [43]. Moreover, FGFR activation can also promote CBL-mediated PI3K degradation, providing an additional layer in FGF signaling regulation by attenuating the Akt/PKB pathway [44]. Further feedback inhibition comes from the induction of Sef (Similar expression to Fgf), Sprouty (SPRY) proteins and MAPK phosphatase 3 (MKP3) [45]. Sef is a transmembrane protein that inhibits the dissociation of the MEK-MAPK/Erk complex, preventing nuclear translocation of activated MAPK/Erk and thus the transcription of effector genes [46]. In addition to attenuate MAPK/Erk signaling, Sef may also interact directly with FGFR through its extracellular domain, preventing receptor autophosphorylation [47]. SPRY proteins and MKP3 also inhibit the MAPK/Erk pathway. While SPRY proteins interact with Grb2 to prevent SOS-mediated Ras activation or bind to Raf to block subsequent activation of downstream targets [45], MKP3 directly dephosphorylates MAPKs (Erk1 and Erk2) [48]. Interestingly, Erk1 and Erk2 also act as negative regulators of FGF signaling by phosphorylating FGFR at the C-terminal portion, inhibiting its tyrosine kinase activity [49] (Fig. 3).

### FGF signaling in axons

#### Axonal specification and growth

The characteristic morphology of the nerve cell relies on the polarization of its neurites, i.e., the formation of a single axon and multiple dendrites. Axonal specification occurs early in development and requires proper regulation of microtubules (MTs) dynamics. Increased MT stabilization induces the formation of a single axon from the multitude of primordial neuronal branches [50]. This is achieved with the help of MT-stabilizing proteins (MSPs) that not only promote MT assembly but also protect MTs from depolymerization [51]. Interestingly, the intracellular FGF13 appears to possess the characteristics of an MSP. FGF13 interacts directly with MTs via a tubulin-binding domain and induces their polymerization and stabilization in the growth cone [52]. Due to its enrichment and function in this particular region of the axon, it is not surprising that loss of FGF13 largely impairs axonal formation and refinement. Most FGF13-deficient neurons fail to develop a single axon, whereas those that are able to acquire a polarized shape develop a unique axon with an aberrant number of branches [52]. Consistently, neurons lacking several members of the MSPs family such as MAP1B or Tau, also exhibit an impairment in axon formation or develop highly branched axons/leading processes [51], reinforcing the role of FGF13 as an MSP, whose interaction with MTs is essential for regulating axonal specification.

After a neurite acquires the identity of an axon, it must grow and extend multiple branches to communicate with several synaptic partners. FGF2, also known as basic FGF, has axon outgrowth and branching activities [27, 53–56]. In dorsal root ganglia neurons (DRGs), these neurotrophic effects appear to be mediated by interaction with FGFR1, since overexpression of this receptor augments FGF2-induced axon outgrowth [53]. Interestingly, the intracellular trafficking of FGFR1 seems to dictate the morphological alterations induced by FGF2 in these neurons. Increasing FGFR1 recycling, through inhibition of its lysosomal degradation or by altering its ubiquitination status, promotes axonal elongation [53, 54]. By contrast, inhibition of FGFR1 endocytosis prevents FGF2-induced axonal outgrowth but enhances axonal branching [57]. However, it should be noted that in central nervous system (CNS) neurons, the mechanism of action of FGF2 may be slightly different. Application of FGF2 promotes mainly interstitial branching of cortical axons by enhancing the pausing and enlargement of their growth cones [55] and it is unknown which FGFR modulates its action. In hippocampal neurons, FGF2 enhances the severing of MTs through expression of the MT-severing proteins katanin and spastin, resulting in axonal branching [58]. This is an important step in branch formation, since local fragmentation of bundled MTs into several very short fragments is required for the entry of MTs into the axonal filopodia where the new branch will arise [59]. Although activation of the MAPK cascade is required for FGF2-induced axonal branching in hippocampal neurons [56], it is unclear whether this pathway mediates the changes in MTs dynamics induced by FGF2. Surprisingly, FGF2 has an inhibitory effect on axonal branching in cultured hippocampal granule neurons [60]. Overall, these findings indicate that the changes in axonal morphology elicited by FGF2 may be cell specific, with different populations of neurons exhibiting distinct responses to FGF2 stimulation.

Unlike FGF2 that decreases the branching level of hippocampal granule cell axons (mossy fibers) in culture, FGF4, FGF5, FGF7 and FGF8 enhances it [60]. Moreover, FGF4-mediated axonal branching requires the participation of the extracellular protein neuritin that facilitates the recruitment of FGFR1 to the axonal surface. Neuritin is upregulated by neural activity and excess amounts of this protein enhances axonal branching in granule neurons through activation of FGFR1 signaling [60]. Since neuritin mRNA is predominantly expressed in the dentate gyrus of the hippocampus [61], these findings raise the possibility that neuritin and FGF4 may cooperate in inducing mossy fiber sprouting during periods of excessive neuronal activity, contributing to the exacerbation of epilepsy. Besides its axon branching activities, FGF8 also promotes neurite outgrowth of cultured cochlear spiral ganglion neurons through FGFR-dependent activation of NFκB signaling pathway [62].

Additional regulation of axonal morphology comes from proteins that modulate FGF signaling. Anosmin-1, the protein defective in the X-linked form of Kallmann syndrome, is one among such proteins. In the olfactory system, this extracellular matrix protein promotes axonal branching of mitral and tufted cells, the olfactory bulb output neurons [63]. A similar effect is also observed in the cerebellum, where anosmin-1 induces growth and branching of Purkinje axons [64]. Although, the molecular pathways that govern these effects in mammals remain poorly understood, in *C.elegans* anosmin-1 branch-promoting activity depends on FGF and involves the formation of a receptor complex with L1CAM and FGFR [65]. SPRY proteins that exert negative feedback control on several elements of the FGFR cascade, have also been implicated in axonal development. Downregulation of Spry2 in DRG neurons, as well as, Spry2 and Spry4 in hippocampal neurons promotes axon outgrowth and enhances FGF2 trophic effects, whereas Spry2 overexpression inhibits axon growth without affecting cell viability [66, 67].

#### Axon guidance

**Fig. 4:**
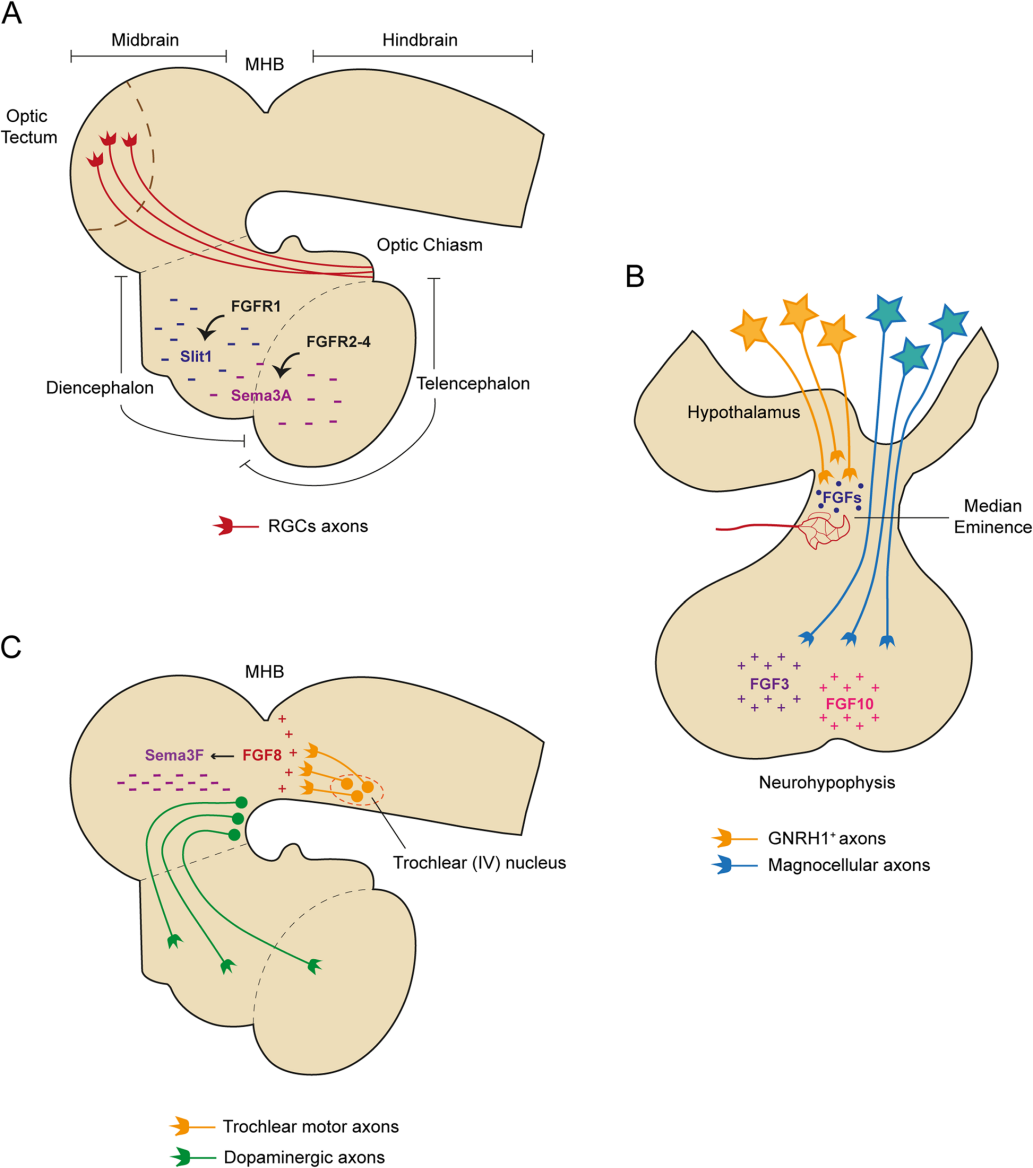
Role of FGF signaling in axon pathfinding. **A** Schematic diagram of the neural tube showing the regulation of the trajectory of RGCs axons by FGF signaling. In the *Xenopus*, slit1 and sema3A expression in the forebrain is positively regulated by FGFR1 and FGFR2-4 respectively. These two guidance molecules work together to repel RGCs axons out of the mid-diencephalon in the direction of the optic tectum (superior colliculus in mammals). **B** Schematic representation of the hypothalamus-hypophyseal system. During development, neurons that synthesize GNRH1 send their axons to the median eminence to ensure the release of GNRH1 into circulation to reach the adenohypophysis (not shown). FGFs emanating from the median eminence may act as chemoattractive cues, since the expression of a dominant negative form of FGFR1 in GNRH1 neurons compromises the targeting of their axons to this region. By contrast, magnocellular axons traverse the median eminence and reach the neurohypophysis where they release the peptides vasopressin and oxytocin into the general circulation. In the chick brain, FGF3 and FGF10 secreted by the neurohypophysis attract these hypothalamic neurosecretory axons towards this region. **C** Side view of the neural tube showing the effect of FGF8 signaling in axon pathfinding. FGF8 produced by the isthmic organizer at the MHB attracts trochlear motor axons as they extend from cell bodies in the anterior hindbrain. This MHB-derived FGF8 also regulates the growth of midbrain dopaminergic axons by inducing the expression of sema3F in the midbrain. This repulsive cue guides dopaminergic axons rostrally towards their diencephalic and telencephalic targets. GNRH1, Gonadotropin-releasing hormone; MHB, Midbrain-hindbrain boundary; RGCs, Retinal ganglion cells

Growing axons require proper guidance to accurately find their targets and establish the synaptic contacts that will define neural circuits. The central component of the axonal navigation system is the growth cone, a dynamic structure at the tip of the extending axon that receives directional information from molecular cues in the environment to drive the axon in the correct direction. Several families of guidance cues (including Netrin, Semaphorin, Slit and Ephrin family) have proven to be essential for the guidance and targeting of axons [68]. However, there is increasing evidence that well-established patterning molecules such as FGFs can also provide chemotrophic cues for growth cones [69]. A prime example comes from *Xenopus laevis*, where the expression of a dominant negative form of FGFR1 in RGC causes anomalies in optic tract development. Dominant negative-expressing axons grow at a slower rate than normal and fail to reach their destination, the optic tectum, indicating that FGF signaling participates in the process of target recognition [70]. Moreover, in dissociated cultures of RGCs from *Xenopus*, FGF2 has a chemorepulsive effect on growth cones, which is mediated by PLC pathway [27]. Besides acting directly on growth cones to mediate their turning, FGFs can also influence axonal navigation indirectly by controlling the normal expression of guidance cues in the developing neuroepithelium. Pharmacological inhibition of FGFR function during *Xenopus* optic tract development results in a rapid downregulation of sema3A and slit1 in the forebrain. As a result, RGC axons fail to navigate through the mid-diencephalon towards the optic tectum [71]. While signaling through FGFR1 maintains forebrain slit1 levels [72], sema3A expression is regulated by FGFR2-4 [73]. Interestingly, all receptors converge on a common intracellular signaling mechanism to regulate the expression of the two guidance molecules that involve the PI3K-Akt pathway [73]. Taken together, these findings indicate that FGF signaling positively regulates the expression of both sema3A and slit1, which work together to repel RGC axons out of the mid-diencephalon in the direction of the optic tectum (Fig. 4A). Regarding the FGFRs ligands that can underlie the observed effect in the *Xenopu*s retinotectal system, FGF8 emerges as a strong candidate, since its overexpression increases slit1 and sema3A levels in several regions of the neuroepithelium [71]. However, further studies should be performed to assess whether the axon guidance defect observed with FGFR inhibition can be replicated in the absence of FGF8 activity.

FGF signaling is also required for the correct wiring of the hypothalamus-hypophyseal system (Fig. 4B). Expression of a dominant negative FGFR1 in murine gonadotropin-releasing hormone (GNRH1) neurons compromises the targeting of their axons to the median eminence, a development process essential for GNRH1 secretion and ultimately fertility [74]. In chick brain explants, FGF3 and FGF10 secreted from the forming neurohypophysis attract hypothalamic neurosecretory axons towards this region. Interestingly, FGF10 shifts its chemoattractant function to repellant activity at high concentrations, suggesting a mechanism for the stalling of axons as they reach the neurohypophysis. In agreement with these results, the zebrafish *fgf3* mutants and transgenic fish expressing a dominant negative FGFR1 in hypothalamic neurons lack neurohypophysis innervation [75].

Within the neural tube, FGFs are expressed in several organizing centers, i.e., signaling centers that instruct the fate, growth and organization of nearby tissues in a position-specific manner. Thus, FGFs are placed in a strategic location to influence the guidance and targeting of axons. In fact, studies in chick embryos and rat brain explants show that FGF8 produced by the isthmic organizer at the midbrain-hindbrain boundary (MHB) attracts trochlear motor axons as they extend from the cell bodies located in the anterior hindbrain [76] (Fig. 4C). On the other hand, MHB-derived FGF8 controls the directed growth of midbrain dopaminergic axons by inducing the expression of the chemorepellent sema3F in the midbrain. This molecular cue prevents dopaminergic axons to grow caudally and invade the hindbrain or to deflect dorsally, guiding them rostrally towards their diencephalic and telencephalic targets [77] (Fig. 4C).

FGFs produced outside the nervous system also work as guidance cues for peripheral axons. In explant cultures, murine spinal motor neurons from the medial class (MMCm) extend their axons towards FGF-producing somites. This attraction is completely blocked by the presence of FGFR inhibitors, demonstrating the involvement of FGFs in the guidance process. The chemoattractant function of FGFs appears to be mediated by FGFR1, since its conditional deletion in transgenic mice results in the misprojection of several MMCm axons [78].

Overall, FGF signaling seems to play an important role in the guidance of axons, whether they project to the periphery or remain within the nervous system. FGFs can either act directly on growth cones to influence their turning or as signaling molecules that regulate the expression of well-established guidance cues in the neuroepithelium.

#### Presynaptic formation

Once the axon has found its target location, presynaptic differentiation ensues in the nerve terminal. This developmental process leads to a well synchronized establishment of new synaptic contacts and neuronal circuits, which requires a coordinated local rearrangement of the pre and postsynaptic sites. Synapse formation and presynaptic organization are strongly dependent on a variety of adhesion proteins, soluble factors and other molecules that act as presynaptic organizers such as laminin β2, neuroligins, ephrins, synCAMs, WNT-7, SIRPs, FGFs and other factors [79, 80]. The first evidence that FGFs might have a role in presynaptic formation arose in 1995 when Benjamin Peng and colleagues showed that beads coated with FGF2 induced presynaptic formation in *Xenopus* spinal cord neurons [81]. Later, Toru Imamura and colleagues showed the ability of FGF2 to increase the number of excitatory synapses in rat hippocampal neurons in a dose-dependent manner, by promoting the clustering of presynaptic vesicles, which in turn colocalize with postsynaptic sites enriched in PSD-95 and GluR1, indicating that these are mature synapses [82, 83]. Addition of a MAPK inhibitor eliminated the effects of FGF2, indicating that MAPK signaling is required for FGF2-induced presynaptic differentiation [82].

The FGF system is also important for neuromuscular junction (NMJ) formation. Umemori and colleagues showed that FGF22 promotes synapse formation in motor neurons by inducing redistribution of synaptic vesicle components, particularly synapsin and SV2. The neurites of FGF22-treated neurons present higher numbers of synaptic vesicle clusters and also show higher levels of activity, as demonstrated by FM1-43 dye measurements [84]. FGF7 and FGF10, members of the same sub-family of FGF22, show similar effects on vesicle clustering. Other FGFs showing effects on synaptic vesicle clustering in motor neuron axons include FGF4/6/9 [84]. FGF7/10/22 are expressed in myotubes and act on FGFR2b present in motor axons to guide the establishment of the NMJ [2, 85]. Neutralization of FGF7/10/22 by a soluble FGFR2b-AP peptide strongly decreased the number of presynaptic varicosities at neurite-myotube contact sites, revealing the importance of these FGF family members on NMJ formation. In addition, mice embryos lacking FGFR2b show reduced levels of synaptic vesicle clustering in motor neurons nerve terminals [85]. However, this reduction is only observed until as late as postnatal day 7, after which there is close to no observable difference in the accumulation of synaptic vesicles in motor neurons nerve terminals. This suggests that other mechanisms independent of FGFR2b might kick in at later stages of development. While FGF7/10/22 expression is very high in early stages of development and decreases with NMJ maturation, FGFBP1 expression assumes the opposite behavior, suggesting a highlighted role of this FGF binding partner in the consolidation of NMJ maturation [40, 84–86]. In agreement with this, Taetzsch and colleagues have demonstrated that loss of FGFBP1 activity retards the aggregation of synaptic vesicles in NMJ [87].

In addition to their role in the presynaptic differentiation and formation of the NMJ, FGF7 and FGF22 are highly expressed in the CA3 region of the hippocampus and are important players in synapse formation in this region. In developmental stages coincident with synaptogenesis, knockout of both FGF7 and FGF22 strongly compromises synapse formation as observed by a decrease in SV2 staining. Moreover, knockout mice for FGF22 or FGF7 exhibit defects in vesicular glutamate transporter 1 (VGLUT1) clustering and vesicular GABA transporter (VGAT) clustering, respectively, implying that FGF22 promotes excitatory presynaptic formation while FGF7 induces inhibitory presynaptic formation in the hippocampus [86]. In fact, and as expected, by subjecting knockout mice to a seizure-inducing protocol, the authors showed that FGF22-deficient mice are resistant to epileptic seizures while FGF7-deficient mice are susceptible to them as a result of imbalanced excitatory/ inhibitory synaptic activity [86]. Haploinsufficiency of FGFR2b in mice and FGFR1b knockout both lead to decreased levels of VGLUT1 and smaller puncta in the CA3 region of the hippocampus at early stages of development. Loss of FGFR2b but not FGFR1b leads to impaired inhibitory synapse formation as seen by a decrease in the levels of VGAT staining in the CA3 region. Moreover, FGF22 requires both FGFR2b and FGFR1b to induce excitatory presynaptic differentiation in cultured hippocampal neurons, while only FGFR2b is required for an inhibitory presynaptic response to FGF7 [88]. Together, these results reveal that distinct sets of FGFRs mediate excitatory and inhibitory synaptogenesis in the hippocampus. Additionally, the authors also show that FGFR2b-mediated FGF22 presynaptic effects are dependent on FRS2 and PI3K signaling, suggesting a potential involvement of the PI3K/Akt pathway downstream of FGFR2b activation [88]. FGF22 is not only a presynaptic organizer, but also contributes to presynaptic terminals stabilization, by promoting the expression of insulin-like growth factor 2, which in turn is a key player in the stabilization of presynaptic terminals [89]. Interestingly, a recent study showed that FGF22-induced synapse formation, and the subsequent axonal maturation significantly reduces the level of ribosomes in distal axons [90]. The authors further show that this loss of ribosomes is selective to axons, as ribosome levels in the cell body are unaffected by synaptogenic signals acting specifically in axons. This decrease in axonal ribosomes is mediated by the ubiquitin–proteasome system (UPS), since MG132 and epoxomicin, two inhibitors of the UPS prevented the FGF22-induced ribosomal decrease [90].

**Fig. 5:**
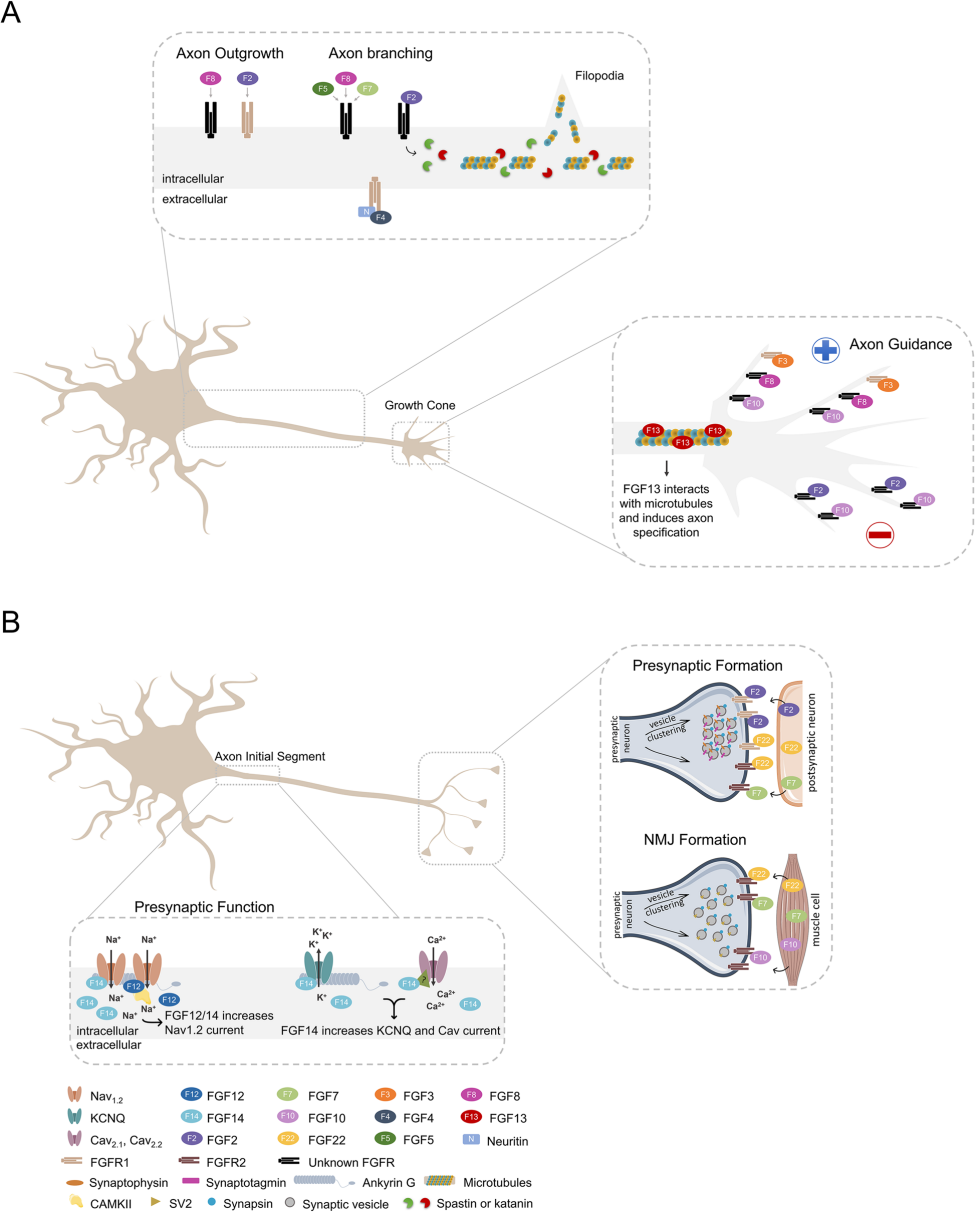
FGFs involved in axonal development and function. **A** Schematic representation of the FGFs/FGFRs that play important roles in axon specification, extension and guidance. In dorsal root ganglia neurons, FGF2 promotes axon outgrowth and branching through activation of FGFR1. In hippocampal neurons, the FGF2 branching activity involves the expression of the MT-severing proteins katanin and spastin that cut bundled MTs into shorter fragments to allow their entry into the axonal filopodia where the new branch will arise. FGF4, FGF5, FGF7, and FGF8 enhance axonal branching in hippocampal granule neurons. The FGF4-mediated axonal branching requires the participation of the extracellular protein neuritin that promotes the recruitment of FGFR1 to the axonal surface. Besides its axon branching activities, FGF8 also promotes axon outgrowth of cochlear spiral ganglion neurons. In the growth cones, FGF13 acts as an MSP, whose interaction with MTs is essential for regulating axonal specification. A subset of FGFs can also provide chemotrophic cues for growth cones. FGF3, FGF8 and FGF10 act as attractive cues, while FGF2 has a chemorepulsive effect on growth cones. At higher concentrations FGF10 shifts its chemoattractant function to repellant activity. **B** FGFs are key in driving presynaptic formation while also playing an important role in the regulation of voltage-gated channels and, consequently, modulating the generation of action potentials. FGF2 acts on presynaptic terminals to induce clustering of synaptic vesicles, namely synaptotagmin, synapsin I and synaptophysin, in *Xenopus* spinal cord neurons and rat hippocampal neurons. Other FGF family members, such as FGF7, FGF10 and FGF22 are expressed in myotubes and act on motor neurons nerve terminals through FGFR2b to induce clustering of synapsin I and SV2 synaptic vesicles. In addition to their role in neuromuscular junction formation, FGF7 and FGF22 are crucial inhibitory and excitatory presynaptic organizers in hippocampal neurons, respectively. While FGF22 signals through FGFR1b and FGFR2b to induce presynaptic differentiation, FGF7 only activates FGFR2b. A subset of intracellular, non-secreted FGFs play important roles in the regulation of voltage-gated channels in the AIS, which are important for proper action potential generation. FGF14 and FGF12 both regulate Nav_1.2_ channels, a highly expressed type of Nav channels in the AIS, the latter through a CAMKII-dependent mechanism. FGF14 regulates the localization and currents of Nav and KCNQ channels in the AIS, while also regulating presynaptic Cav_2.1_ and Cav_2.2_ channels. CAMKII, calmodulin-dependent protein kinase II; KCNQ, Kv channels**;** MSP, microtubule-stabilizing protein; MTs, microtubules

These studies show that the FGF system intervenes in different steps of presynaptic differentiation in the peripheral nervous system (PNS) and CNS to promote the formation of a functional presynaptic terminal (Fig. 5).

#### Presynaptic function

Once presynaptic and postsynaptic elements have cooperated in the formation of a functional synaptic contact, pre and postsynaptic neurons are ready for proper neuronal communication. The axon initial segment (AIS) comprises the initial section of the axon and is an essential structure in the initiation of action potentials, which will in turn propagate throughout the axon. Clustering of voltage-gated sodium (VGSC) and potassium (KCNQ) channels in the AIS is key for effective action potential initiation and these channels are regulated by a subset of intracellular FGFs [91–93]. FGF14 is highly expressed in the proximal region of the AIS but has low expression in the somatodendritic compartment [92]. Interestingly, FGF14 knockdown decreases VGSCs signal and current density [92, 94]. Unlike what stands true for Nav channels, FGF14 does not directly bind to Cav2 channels [11, 95]. Both FGF14 knockdown and FGF14bF150S, a missense mutation, reduce calcium channel currents in granule neurons [11], similarly to effects on Nav channels [94]. FGF14 regulation of Cav channels appears to be specific for presynaptic calcium channels, Cav2.1 and Cav2.2, with no effect on somatodendritic calcium channels, Cav2.3 and Cav1.2. FGF14 also regulates the subcellular localization and activity of KCNQ2 channels in the AIS in hippocampal neurons. FGF14 knockdown in hippocampal neurons leads to a decrease in the levels of KCNQ2 in the AIS as well as to a decrease in the KCNQ currents. When co-transfected with KCNQ2 in HEK cells, FGF14 is co-immunoprecipitated with KCNQ2, revealing an interaction between the two. FGF14 regulates both KCNQ and VGSC channels independently, as in a heterologous system regulation of KCNQ channels by FGF14 is not dependent on the presence of VGSC [91].

FGF13 however is expressed both in the somatodendritic compartment and in the proximal and distal region of the AIS [92, 96]. FGF13 knockdown increases surface VGSC levels and total VGSC current, but not axial current and VGSC levels in the AIS, suggesting that FGF13 regulates the somatodendritic VGSC by limiting their surface expression and has no influence on the AIS-localized VGSC [92]. Thus, FGF13 and FGF14 act in concert to assure the correct maintenance of VGSC surface expression higher in the AIS than in the somatodendritic compartment, in order for proper action potential generation.

FGF12 also localizes to the AIS. Laezza and colleagues performed affinity purification of the Nav1.2 channel followed by mass spec analysis. FGF12 was found to be one of the interacting proteins that compose the Nav1.2 complex [97], indicating that FGF12 is a component of the Nav1.2 channel complex. The authors further showed that FGF12 modulates Nav1.2-encoded currents together with calmodulin-dependent protein kinase II (CaMKII), in a phosphorylation-dependent manner [97]. Contrary to the previous study, Wang and colleagues showed an effect of FGF12b isoform in shifting the V1/2 of inactivation of Nav1.2 channel [98]. Furthermore, granule neurons from mice lacking both FGF12 and FGF14 show impaired Nav channel inactivation, as well as compromised recovery from negative membrane potential and overall neuron excitability compromised [96]. Altogether, these observations show that FGF12, FGF13 and FGF14 are localized in the axon initial segment and regulate the accumulation and/or function of voltage-gated channels in this axonal region and have a potential role as intrinsic modulators of neuronal excitability (Fig. 5).

#### The FGF system and disease

Given their widespread involvement in neural development, it is not surprising that dysregulation of the FGF system underlies several neurological conditions. Indeed, FGFs have been linked to psychiatric disorders like depression and schizophrenia and neurodegenerative diseases, such as Parkinson’s disease, Alzheimer’s disease and multiple sclerosis [99]. Because this review examines the involvement of FGF signaling in axon development and function (Table 2), we will focus this section on how aberrant FGF signaling can contribute to the pathogenesis of specific neurological conditions due to axon-related alterations. Next, we will focus our attention on the possible involvement of FGFs in axon regeneration/repair in both the PNS and CNS.

**Table 2:** FGF signaling during axon development and function

*FGFs*	*FGFRs*	*Signaling Pathways*	*Role in axon*	*Biological Function*	*Model*	*Ref*
FGF2	FGFR1	**-**	Axon outgrowth/Axon branching	Promotes axonal elongation and branching in cultured DRGs neurons	Rat	[53, 54, 57]
	-	-	Axon branching	Promotes mainly interstitial branching of cortical axons in vitro	M.auratus	[55]
	-	MAPK/Erk (?)	Axon branching	Enhances the severing of MTs in hippocampal neurons through expression of katanin and spastin	Rat	[58]
	-	-	Inhibition of axon branching	Suppresses axonal branching in cultured hippocampal granule neurons	Rat	[60]
	-	PLC	Axon guidance	Chemorepulsive effect on RGCs growth cones	Xen	[27]
	-	-	Presynaptic formation	Induces presynaptic vesicle clustering	Xen/Rat	[81, 82]
FGF3	FGFR1 (?)	-	Axon guidance	Attracts hypothalamic axons towards the neurohypophysis	Chick/Zeb	[75]
FGF4	FGFR1/Neuritin	-	Axon branching	Enhance axonal branching of cultured hippocampal granule neurons	Rat	[60]
FGF5	-	-				
FGF7	-	-				
	FGFR2b	-	NMJ formation	Promotes NMJ formation by increasing vesicle clustering in motor neurons	Mouse	[84, 85]
	FGFR2b	-	Presynaptic formation	Promotes inhibitory synapse formation in hippocampal neurons by increasing synaptic vesicle clustering	Mouse	[86]
FGF8	-	NF_K_B	Axon outgrowth	Promotes neurite outgrowth of cultured cochlear spiral ganglion neurons	Mouse	[62]
	-	-	Axon branching	Promotes axonal branching of culture hippocampal granule neurons	Rat	[60]
	-	-	Axon guidance (?)	Induces the expression of slit1 and sema3A	Xen	[71]
	-	-	Axon guidance	Attracts trochlear motor axons from the hindbrain towards the isthmic organizer	Chick/Rat	[76]
	-	-	Axon guidance	Regulates growth of midbrain dopaminergic axons by inducing sema3F	Rat	[77]
FGF10	-	***-***	Axon guidance	Regulates growth of hypothalamic axons towards the neurohypophysis	Chick	[75]
	FGFR2b	-	NMJ formation	Promotes NMJ formation by increasing vesicle clustering in motor neurons	Mouse	[84, 85]
FGF12	-	***-***	Action potential generation	Regulates AIS-localized VGSC currents	Rat	[97]
FGF13	-	***-***	Axon specification	Induces MTs polymerization and stabilization in the growth cone	Rat/Mouse	[52]
FGF14	-	***-***	Action potential generation	Regulates AIS-localization and currents of VGSC and KCNQ channels	Mouse/Rat	[91]
	-	***-***	Action potential generation	Regulates presynaptic calcium channel currents	Mouse/Rat	[94]
FGF22	FGFR2b	-	NMJ formation	Promotes NMJ formation by increasing vesicle clustering in motor neurons	Mouse	[84, 85]
	FGFR2b	PI3K/Akt (?)	Presynaptic formation	Promotes excitatory synapse formation in hippocampal neurons by increasing synaptic vesicle clustering	Mouse	[86, 88]
	FGFR1b	-				
-	FGFR1	PI3K-Akt	Axon guidance	Regulates slit1 levels in the forebrain	Xen	[72, 73]
-		-	Axon guidance	Required for the targeting of GNRH1 axons to the median eminence	Mouse	[74]
-		-	Axon guidance	Required for the growth of MMCm axons towards FGF-producing somites	Mouse	[78]
	FGFR2-4	PI3K-Akt	Axon guidance	Regulate sema3A expression in the forebrain	Xen	[73]

*AIS* Axon initial segment, *DRG* Dorsal root ganglia, *GNRH1* Gonadotropin-releasing hormone, *KCNQ* Voltage-gated potassium channels, *M.auratus Mesocricetus auratus*, *MTs* Microtubules, *MMCm* Spinal motor neurons from the medial class, *NMJ* Neuromuscular junction, *RGC* Retinal ganglion cell, *Sema* Semaphorin, *VGSC* Voltage-gated sodium channels, *Xen Xenopus laevis*, *Zeb* Zebrafish

### Neurological diseases

Dysregulation of FGF signaling has been associated with ictogenesis, the generation of a seizure. For example, overexpression of FGF2 in mice makes them more vulnerable to kainate-induced seizures. Given the role of FGF2 in excitatory synapse formation in cultured hippocampal neurons, this increased susceptibility to chemical-induced seizures in transgenic animals may arise from an increase in excitatory synaptic transmission. In fact, FGF2 overexpression increases the density of glutamatergic presynaptic sites in different areas of the hippocampus and the number of excitatory inputs in the CA1 region, suggesting the presence of a latent hyperexcitability. Interestingly, it reduced seizure-induced cell death, probably due to a well-characterized neuroprotective role of this pleiotropic growth factor [100]. The intracellular FGF13 that regulates the surface expression and currents of VGSC, also plays a part in the control of neuronal excitability in the hippocampus. Heterozygous female mice in which one FGF13 allele is deleted exhibit increased sensitivity to hyperthermia-induced seizures and spontaneous recurrent seizures. This phenotype arises from altered excitatory and inhibitory synaptic inputs to CA1 pyramidal cells. FGF13 mutant mice display a decrease in inhibitory and an increase in excitatory transmission in the CA1 region when compared to wild-type mice. Whether the observed hyperexcitability results from altered VGSC physiology remains to be elucidated. Interestingly, a maternally transmitted balanced translocation between chromosomes X and 14 that disrupts FGF13 gene was identified in a family with a common genetic epilepsy syndrome know as Genetic Epilepsy with Febrile Seizures Plus, suggesting that mutations in FGF13 that result in an unusual phenotype of neuronal hyperexcitability, may contribute to the pathogenesis of epileptic disorders [101]. Moreover, as previously mentioned, knockout of the inhibitory presynaptic organizer FGF7 in mice also increases seizure susceptibility. Taken together, these results show that alterations in FGF signaling affect the neuronal connectivity in the hippocampus which may contribute to epileptogenesis.

Lessening FGF22 activity causes a depressive-like behavior in mice [102]. This phenotype seems to be due to the reduced number of excitatory synapses formed onto CA3 pyramidal neurons. Specific FGF22 inactivation in the mouse CA3 region compromises local excitatory synaptogenesis and induces a depressive-like phenotype similar to the full knockout [103]. These results indicate that CA3-derived FGF22 contributes to the establishment of synaptic circuits involved in affective behavior and that dysregulation of its signaling during development can increase the propensity for depression.

### Axonal injury and regeneration

In the PNS, axons show a remarkable ability to repair themselves after injury, while neurons within the CNS do not spontaneously regenerate. Axonal regeneration is a complex process that involves distinct multicellular responses and a spatiotemporal regulation of several growth factors and cues to create the appropriate milieu for regeneration to occur [104]. Since FGFs regulate several aspects of axonal development and function, their signaling may also be important to promote the regrowth of injured axons. In fact, some FGFs are strongly and rapidly up-regulated in response to peripheral nerve lesions. Increased expression of FGF2 is observed in the rat hypoglossal nucleus and nerve as well as in sympathetic ganglia following nerve injury [105, 106]. FGF2 and FGFR3 mRNAs are also up-regulated in the rat sciatic nerve and corresponding ganglia after crush, while FGF1 and FGF2 protein levels increase in the proximal and distal segments of the transected sciatic nerve [107, 108]. These findings suggest an involvement of endogenous FGF signaling in nerve regeneration. Notably, FGF1 and FGF2 have both been shown to improve the regeneration of damaged nerves. Administration of FGF1 to the rat vagus nerve or FGF2 to the mental nerve after a crush injury facilitates axonal regeneration [109, 110]. In fact, substantial data have been gathered demonstrating the relevance of FGF2 in peripheral nerve regeneration. Schwann cells overexpressing the high molecular weight FGF2 isoforms (21/23 kDa) transplanted into the injured sciatic nerve enhance axon regeneration and mediate early recovery of sensory function [111, 112]. The low molecular weight FGF2 isoform (18 kDa) released by grafted Schwann cells also improves the regeneration of sensory and motor axons and consequent reinnervation of hindlimb muscles following sciatic nerve injury [113]. Moreover, transgenic mice expressing high levels of FGF2 exhibit faster axon regeneration and an increased number of Schwann cells in the distal part of the crushed sciatic nerve [114]. This mitogenic effect on Schwann cells raises the possibility that FGF2 may promote axon regeneration indirectly by increasing the number of Schwann cells in the distal stump, which in turn support the regrowth of regenerating axons through the release of trophic factors and expression of cell adhesion molecules [104]. FGF2 also appears to be required for the proper reinnervation of muscle fibers after nerve injury. Mice lacking FGF2 exhibit an increase in polyneuronal innervation at neuromuscular synaptic sites after facial nerve injury, delaying the restoration of function [115]. Further studies should be performed to clarify the precise role of FGF2 in peripheral axon regeneration and in the reestablishment of the neuromuscular junction after injury. Observations made in Spry2-deficient mice corroborate the positive effects of FGF signaling in peripheral regeneration. Following sciatic nerve crush, heterozygous Spry2 knock-out mice recover faster their motor function than wild-type mice as a consequence of higher numbers of regenerating myelinated axons and increased expression of the regeneration-associated protein GAP43 in the injured nerve [116].

Enhanced FGF signaling has also been shown to promote optic nerve regeneration. In mammals, the axons of adult RGCs do not normally regenerate after optic nerve injury. However, overexpression of FGF2 in rat RGCs through virally-mediated gene delivery is able to promote axon regrowth across the injury site [117, 118]. This regenerative effect is mediated by the MAPK/Erk pathway, since pharmacological inhibition of this signaling cascade markedly inhibited FGF2-induced RGC axon regeneration [118]. Both FGFR1 and heparan sulfate are expressed by adult rat RGCs [117], suggesting that the regenerative response to FGF2 up-regulation can be mediated by autocrine signaling. However, endogenous FGF2 is not present in mature RGC bodies and axons, only in glial cells of the ganglion cell layer, inner nuclear layer and optic nerve [117, 119]. Although injury to the optic nerve increases endogenous FGF2 expression within the retina, this occurs mainly in the outer nuclear layer and not in RGCs [119]. Therefore, this injury-mediated upregulation of endogenous FGF2 is insufficient to reproduce the axonal outgrowth effects induced by the FGF2 gene delivery. Interestingly, in the amphibian visual system, which is able to repair itself, FGF2 and all four FGFRs are present in adult RGCs and their pattern of expression in the retina and optic tectum change after optic nerve injury. FGF2 expression increases in the cell bodies of the ganglion cell layer and inner nuclear layer shortly after injury. Axotomy also induces a prolonged upregulation of FGFR1 and FGFR3 in the retina, particularly in the ganglion cell layer [120]. Moreover, administration of FGF2, either directly to the crushed nerve or intraocularly, improves axon regeneration in the frog optic nerve through activation of FGFR1 [121]. These findings support the idea that endogenous FGF2 signaling may play a part in the spontaneous regeneration of the amphibian visual system following injury. Overall, FGF2 emerges as a promising target to explore in future strategies seeking to repair the mammalian optic nerve.

Injury to the spinal cord results in severe neurological impairments because axons fail to regenerate and reestablish lost synaptic contacts. However, some functional restoration can be achieved in incomplete lesions due to the remodeling of spinal and supraspinal axonal circuits. For example, in response to a thoracic transection, injured corticospinal tract (CST) axons sprout into the gray matter of the cervical spinal cord and establish new synapses with long propriospinal neurons that act as relays to lumbar motor circuits [122]. The formation of this detour circuit, that allows the functional communication between the motor cortex and the lumbar spinal cord, is highly dependent on FGF22 signaling. This presynaptic organizer is present in adult spinal interneurons, including the long propriospinal neurons, while its two main receptors, FGFR1 and FGFR2, are expressed by CST axons. FGF22 deficiency or the targeted deletion of both receptors in the mouse motor cortex compromises the formation of new synapses between the CST collaterals and relay neurons in the injured spinal cord. As a result, the detour circuit is not properly formed limiting functional recovery [123]. These findings reveal FGF22 as an important mediator of synapse formation and circuit remodeling after injury, suggesting that the developmental mechanisms that instruct synapse formation in the CNS can be reactivated to favor regeneration and functional recovery.

Strategies based on enhancing FGF signaling to repair the injured spinal cord, have also been employed. The intracellular FGF13, which seems to possess the characteristics of an MSP, is strongly up-regulated in the rat spinal cord shortly after injury and then it gradually returns to basal levels. Overexpression of FGF13 in the injury site, through virally-mediated gene delivery, promotes a significant increase in the number of regenerating CST axons that reach the proximal injury border. This regenerative effect is probably due to an increase in MT stabilization in injured axons, since the levels of acetylated-tubulin increased in the spinal cords of FGF13-overexpressing animals. Although FGF13 was unable to induce robust CST axon regeneration across the injury site, FGF13-overexpressing animals display better motor performances [124]. In contrast to FGF13, endogenous FGF1 levels decrease in the rat spinal cord following injury [125]. Augmenting its expression, through virally-mediated gene transfer, promotes functional recovery via neuroprotection, axon regeneration and remyelination [125, 126]. Moreover, a clinical trial to evaluate the effect of FGF1 on the neurological improvement of patients with subacute or chronic spinal cord injury (SCI) was also performed (Clinical Trials NCT03229031). FGF1 was administered to 46 patients (21 cervical- and 25 thoracolumbar-SCI) three times: once directly to the injured tissue during neurolysis surgery and twice via lumbar punctures three and six months later. The patients were monitored for 48 months and their functional outcomes were evaluated with standardized measurements of neurological function. FGF1 treatment significantly improved the motor scores, sensory scores and functional independence of the patients without major adverse effects [127]. Although no complete recovery of function was observed, this trial reveals promising effects of FGF1 on human SCI and suggests its potential use in combined strategies to restore the injured cord.

FGF2 has also been studied in the context of SCI. Subcutaneous application of FGF2 to mice for 2 weeks after spinal cord hemisection promotes a significant improvement on motor function. The observed beneficial effect appears to be related to an attenuation of inflammation and astrogliosis rather than a trophic effect on injured axons. FGF2 treatment decreases the levels of TNF-α and chondroitin sulphate proteoglycans at the lesion site, gliosis and monocyte/macrophage infiltration [128]. Moreover, astrocytes acquire a bipolar shape with elongated processes along which axons grow and cross the injury site [128]. These FGF-mediated glial cell bridges are also observed in zebrafish that have the capacity to regenerate their spinal cord after injury. Loss of FGF function in zebrafish inhibits the formation of these bridges, preventing axon regeneration after SCI [129]. Together, these results suggest that FGF2 is capable of modulating the inhibitory environment that is created after SCI and to drive changes in glial morphology to bridge the gap of the injured area to support axonal regeneration across it.

## Concluding remarks

Overall, increasing evidence demonstrates that FGF signaling operates in axons from the beginning of their differentiation to the establishment of functional presynaptic boutons. Over 10 members of this vast family of growth factors exhibit axon-related activities, either by acting through FGFRs to activate downstream effectors or intracellularly to directly modulate MTs dynamics or voltage-gated channels. In addition, FGF signaling also influences axonal development indirectly, by controlling the expression of other signaling molecules in the neuroepithelium such as slits or semaphorins. The fact that the same FGF promotes different outcomes in distinct populations of neurons reflects the functional diversity of the FGF signaling system. The different isoforms of the FGFRs, the high diversity of FGFRs coreceptors and modulators and the several signaling cascades activated downstream of FGFRs help to broaden the range of cellular responses to FGFs. The mechanisms by which FGFs govern each step of axonal development remain largely unknown. Elucidating the spatial and temporal expression/regulation of FGFs and their receptors during neurodevelopment may help shed light into this question. Moreover, several studies suggest that interfering with FGF signaling in axons induces alterations in neuronal connectivity that recapitulate key features of neurological disorders. Although these processes are only just beginning to be investigated, they open the possibility that FGF dysfunction in axons may contribute to the pathogenesis of human diseases. Interestingly, FGFs are also critical regulators of synapse formation and maturation during post-injury remodeling of the spinal cord, implying that the developmental mechanisms that direct synapse formation in the CNS can be reactivated after injury. Finally, the fact that endogenous FGF signaling seems to be involved in the regeneration of peripheral nerves and in the spontaneous regeneration of the optic nerve and spinal cord in lower vertebrates, suggests the potential use of FGFs in combined strategies to promote the regrowth of injured axons in the damaged CNS.

## Data Availability

Not applicable.

## Abbreviations

FGF: Fibroblast growth factor
FGFR: Fibroblast growth factor receptor
HSPG: Heparan sulfate proteoglycan
ER: Endoplasmic reticulum
PIP_2_: Phosphatidylinositol-4,5-biphosphate
FRS2: FGFR substrate 2
Grb2: Growth factor receptor-bound 2
SOS: Son of sevenless
MAPK: Mitogen-activated protein kinase
Gab1: Grb2-associated binding protein 1
PI3K: Phosphatidylinositol-3-kinase
PKB: Protein kinase B
PLCγ: Phospholipase Cγ
SH2: Src homology 2
IP_3_: Phosphatidylinositol-3,4,5-triphosphate
DAG: Diacylglycerol
PKC: Protein kinase C
RGCs: Retinal ganglion cells
STAT: Signal transducer and activator of transcription
NLS: Nuclear localization signal
FGFBP: FGF binding protein
FLRT3: Fibronectin-leucine-rich transmembrane protein 3
L1CAM: L1 cell adhesion molecule
Sef: Similar expression to FGF
SPRY: Sprouty protein
MKP3: MAPK phosphatase 3
MTs: Microtubules
MSP: Microtubule-stabilizing protein
DRGs: Dorsal root ganglia neurons
CNS: Central nervous system
GNRH: Gonadotropin-releasing hormone
MHB: Midbrain-hindbrain boundary
MMCm: Murine spinal motor neurons from the medial class
NMJ: Neuromuscular junction
VGLUT1: Vesicular glutamate transporter 1
VGAT: Vesicular GABA transporter
UPS: Ubiquitin-proteasome system
PNS: Peripheral nervous system
AIS: Axon initial segment
VGSC: Voltage-gated sodium channels
KCNQ: Voltage-gated potassium channels
CaMKII: Calmodulin-dependent protein kinase II
CST: Corticospinal tract
SCI: Spinal cord injury
